# Amifostine Suppresses the Side Effects of Radiation on BMSCs by Promoting Cell Proliferation and Reducing ROS Production

**DOI:** 10.1155/2019/8749090

**Published:** 2019-01-09

**Authors:** Bo Huang, Tao He, Qianqian Yao, Liang Zhang, Yang Yao, Hua Tang, Ping Gong

**Affiliations:** ^1^State Key Laboratory of Oral Diseases, West China Hospital of Stomatology, Sichuan University, Chengdu 610041, China; ^2^Dental Implant Center, West China Hospital of Stomatology, Sichuan University, Chengdu 610041, China; ^3^Oral Medical Center, The Second Xiangya Hospital, Central South University, Changsha, China

## Abstract

This study is aimed at investigating the effect of amifostine (AMI) on rat bone marrow stromal stem cells (BMSCs) exposed to 2 Gy radiation. The BMSCs were divided into four groups, namely, group A that received 0 Gy radiation, group B that received 0 Gy radiation and AMI, group C that received 2 Gy radiation, and group D that received 2 Gy radiation and AMI. The proliferation, apoptosis, and distribution of BMSCs in the cell cycle, along with their osteogenesis ability, adipogenesis ability, and ROS production, were subsequently examined. The levels of ALP, PPAR*γ*, P53, and TNF*α* were determined by Western blotting. The results demonstrated that the proliferation of BMSCs and the levels of ALP in group C were much lower than those in group A. The production of ROS and levels of PPAR*γ*, P53, and TNF*α* in the group that received 2 Gy radiation were much higher than those in group A. Furthermore, the production of ROS and the levels of PPAR*γ*, P53, and TNF*α* were much lower in group D than in group C. Additionally, the levels of ALP and extent of cell proliferation were much higher in group D than in group C. The results demonstrated the potential of AMI in reducing the side effects of radiation in BMSCs and in treatment of bone diseases caused by radiation.

## 1. Introduction

Radiotherapy (RT) is an effective strategy for the treatment of tumors, which directly kills tumor cells by inhibiting their proliferation and by inducing apoptosis [1]. At the same time, it damages the surrounding tissues and causes systemic metabolic disorder by increasing the production of reactive oxygen species (ROS) and inflammatory cytokines [2, 3]. ROS and inflammatory cytokines induce cell cycle arrest and trigger mutagenesis, DNA damage, apoptosis, and nucleotide excision repair. These postradiation effects can lead to osteopenia and radiation-induced osteoporosis, thus decreasing bone strength and increasing the risk of serious fractures [4, 5]. The rate of rib fracture increases by ten times in breast cancer patients receiving radiotherapy, in comparison to normal individuals, with the incidence of fractures being as high as 22% in breast cancer patients and 24% in patients with soft tissue sarcomas [4–6]. In addition, the healing time for postradiation fractures in patients with carcinomas is usually more than 6 months and the union is delayed in as many as 67% of the patients [7]. Clinical data have demonstrated that the failure rate of dental implants in irradiated bones is two to three times higher than that in nonirradiated bones [8].

Radiation-induced damages last for a long time, and there exists a possibility of the occurrence of osteoradionecrosis even ten or twenty years after radiation. Although the side effects of radiotherapy have been studied for a long time, a consensus solution to the problem is yet to be found and the prediction of prognosis continues to be difficult [9]. Hyperbaric oxygen (HBO) has often been used to treat the side effects of radiation, since it aids in the diffusion of oxygen to the surrounding tissues, thus improving bone formation and maturation and promoting soft tissue healing. However, HBO therapy is contraindicated in several ailments, including pulmonary diseases, ocular aneurysm, and convulsions associated with oxygen toxicity and rupture of eardrum [10, 11]. However, other studies have reported that HBO does not have additional benefits in improving the success rate of dental implants in irradiated bones [11].

Recent studies have demonstrated that amifostine (AMI; ethanethiol, 2-[(3-aminopropyl)amino]dihydrogen phosphate) could effectively promote the rate of healing in bone fractures, shorten the healing time of fractures, and increase the bone mass in irradiated bones [12, 13]. AMI, first developed by the Walter Reed Army Institute of Research of the US Army, to protect their soldiers from radioactive fallouts, is rapidly dephosphorylated by alkaline phosphatase (ALP), which transforms AMI to its clinically active metabolite, WR-1065. Once activated, AMI protects cells from radiation-induced DNA damages by preventing cellular interactions with DNA radicals and radiation-induced scavenging oxygen free radicals and by donating hydrogens to repair the existing DNA damage [14–17]. The most significant role of AMI in the clinical scenario is its differential effects on cancer cells and normal cells, which attribute to the higher pH and therefore higher activity of ALP in normal cells than in cancer cells, leading to an increased vascular permeation in normal tissues, which activates AMI [17–19]. In the distraction osteogenesis model of murine mandible, it was observed that although the regenerate bone mineral density (BMD) was significantly diminished by radiation, pretreatment with AMI did not only preserve the regenerate BMD but could also greatly promote bone value fraction beyond the normal regenerate density [20].

Although AMI plays an important and positive role in regulating the remodeling of irradiated bones, the mechanism by which AMI affects bone marrow stromal stem cells (BMSCs) and the underlying basis of the role of AMI remains unknown. The aim of this study was to investigate whether AMI can reduce the damages induced by radiation in BMSCs and improve their osteogenic capability. We hypothesized that AMI can mitigate the deleterious effects of radiation on BMSCs and can promote their osteogenic capability, in addition to inhibiting their adipogenic capability by reducing the production of ROS and the levels of inflammatory molecules induced by radiation.

## 2. Materials and Methods

### 2.1. Cell Culture

Eighteen SD rats, weighing 120 ± 10 g, were supplied by Sichuan University Animal Center. Bone marrow cells of the tibiae and femur were flushed out with Dulbecco's modified Eagle medium (DMEM; HyClone, USA). The cells were then cultured in DMEM supplemented with 10% fetal bovine serum (FBS; Gibco, Australia) and 1% penicillin-streptomycin solution (HyClone, USA) and incubated at 37°C in an atmosphere of 5% CO_2_. The nonadherent cells were discarded after twelve hours, and fresh complete medium was added. The medium was replaced every 3 days. The cell expansion (1 : 3) was measured once the cells reached a confluence of 80–90%. Cells in passage 3 (P3) and passage 4 (P4) were used for the subsequent experiments. The isolated cells were identified as marrow cells (MSCs) by their multiple differentiation potential. The cells were divided into four groups: group A that received 0 Gy radiation, group B that received 0 Gy radiation and 10^−7^ M AMI, group C that received 2 Gy radiation alone, and group D that received 2 Gy radiation in conjunction with 10^−7^ M AMI. AMI was administered 30 min prior to radiation.

### 2.2. Cell Proliferation Assay

The P3 cells were digested with 0.25% trypsin. The cells were then seeded on 96-well plates at a density of 1 × 10^4^ cells per well, with different concentrations of AMI (0 M, 10^−5^ M, 10^−7^ M, and 10^−9^ M). The proliferation of the BMSCs was measured on the first, third, fifth, and seventh days by the Cell Counting Kit assay (CCK-8, Dojindo, Japan). The optical density (OD) was measured at 450 nm using a microplate reader (Varioskan Flash, Thermo Fisher Scientific, USA). The proliferation of the cells in the four groups, A, B, C, and D, was determined by the same method.

### 2.3. Radiation

The cells were digested with 0.25% trypsin and resuspended in DMEM. A single dose of 2 Gy gamma radiation was administered at a rate of 0.83 Gy/min in the Seventh People's Hospital in Chengdu, China. The field size was 10 × 10 cm^2^, and the source-bottle distance was 80 cm. The ^60^Co isotope was used as the source of *γ*-rays. The samples in the control setup were kept outside the room, under the same conditions.

### 2.4. Osteogenic Differentiation, ALP Activity Assay, and Adipogenic Differentiation

The cells were digested with 0.25% trypsin and resuspended in DMEM. The cells in the four groups were then seeded in 6-well plates at a density of 2 × 10^4^ cells per well. After 12 hours, the culture medium was replaced by osteogenic medium or adipogenic medium. On the 21st day, the cells were fixed in 90% ethanol for 20 min followed by staining with 1% Alizarin Red (Sigma, USA) for measuring the osteogenic differentiation. The adipogenic differentiation was measured on the 7th day by fixing the cells in 90% ethanol for 20 min, followed by staining with 0.3% Oil Red O (Sigma, USA). Each experiment was performed in triplicate. The images were obtained with a reverse phase contrast microscope (Leica ZE4 HD, high definition, Germany) and analyzed by Image-Pro Plus 6. The quantity of calcium was measured by using cetylpyridinium chloride (CPC). The quantity of triglyceride (TG) in the cells was assessed by the serum TG determination kit (Sigma, USA). For measuring the activity of ALP, the cells were collected on the seventh day, washed twice with cold PBS, and lysed by a freezing-thawing method and ultrasound pyrolysis, the latter being performed three times. The activity of ALP was measured by the ALP activity kit (Nanjing Jiancheng Bioengineering Research Institute, China). The total amount of protein in the cells was measured by the bicinchoninic acid (BCA) protein measurement kit (KeyGen Biotech, China).

### 2.5. Cell Cycle and Apoptosis Assays

The cell cycle was studied by a flow cytometer (Cell Cycle Detection Kit, KeyGen Biotech, China), according to the manufacturer's instructions, and a fluorescent microscope (Cytomics FC 500, Beckman, USA) equipped with an FITC and DAPI filter. Apoptosis was measured by using the Annexin V-FITC/PI Kit (KGA108-1, KeyGen Biotech, China), according to the manufacturer's instructions. Briefly, twenty-four hours following treatment, 5 × 10^5^ cells were digested with 0.25% trypsin, washed with cold PBS, and resuspended in 300 *μ*l of 1x binding buffer containing 5 *μ*l Annexin V-FITC, followed by dark incubation for 10 min at 37°C. PI (5 *μ*l) was added to each sample for coincubation for an additional 5 min. Following incubation, 200 *μ*l of 1x binding buffer was added to resuspend the cells and a minimum of 10^6^ cells were measured by a flow cytometer (FL1 and FL2 emission filter). The value of Q1 indicated the percentage of necrotic/dead cells, while the value of (Q2 + Q3) indicated the percentage of apoptotic cells and Q4 indicated the percentage of normal cells.

### 2.6. ROS Assay

The BMSCs were seeded at a density of 2 × 10^4^ cells on 24-well plates, maintained in triplicate. The production of ROS was measured two hours postradiation, according to the manufacturer's instructions. The intracellular ROS was studied by incubating with DCFH-DA (Sigma, USA) for 30 min. The cells were then gently washed thrice with PBS. The images were obtained with a reverse phase contrast microscope (Leica ZE4 HD, high definition, Germany) and analyzed by the Image-Pro Plus 6 software.

### 2.7. Cell Attachment

The BMSCs were seeded at a density of 1 × 10^5^ cells on a 24-well plate, maintained in triplicate. The number of cells attached to the surfaces was determined 4 hours after radiation, by a scanning electron microscope (SEM, Hitachi S3400 + EDX, KEKY 2800, Japan). Five visual fields were considered for each parallel well.

### 2.8. Western Blot Analysis

The total cellular protein was extracted on the fifth day by lysing the cells on ice, using a lysis buffer (KeyGen total protein extraction kit, KeyGen Biotech, China). After boiling for 5 min, 50 mg of protein was separated by SDS-PAGE on a 10% polyacrylamide gel, at 60 V/cm^2^ for 60 min and at 100 V/cm^2^ for 80 min. The relevant gel bands were subsequently cut and transferred to a polyvinylidene difluoride (PVDF) membrane (Millipore Corp., Bedford, MA). The membranes were first blocked and then incubated overnight at 4°C with anti-ALP antibody (1 : 500, ab83259, Abcam), anti-TNF*α* antibody (1 : 1000, ab220210, Abcam), anti-P53 antibody (1 : 1000, ab26, Abcam), and anti-PPAR*γ* antibody (1 : 1000, ab209350, Abcam). After incubating with HRP-conjugated secondary antibody (1 : 2000, Aviva Systems Biology, China) for 1 hour, the reactive bands were visualized with an enhanced chemiluminescence (ECL) kit (Millipore, Billerica, MA). The results were analyzed by a densitometer (Quantity One, Bio-Rad).

### 2.9. Statistical Analyses

All the statistical analyses were performed with SPSS version 17.0 (SPSS Inc., Chicago, IL). The statistically significant differences were assessed by one-way analysis of variance (ANOVA) and Newman–Keuls post hoc tests. All the data were expressed as the mean ± SEM, and a *P* value < 0.05 was considered statistically significant. Four to five independent replicates were considered for each experiment.

## 3. Results

### 3.1. Characterization of Rat BMSCs

**Figure 1:**
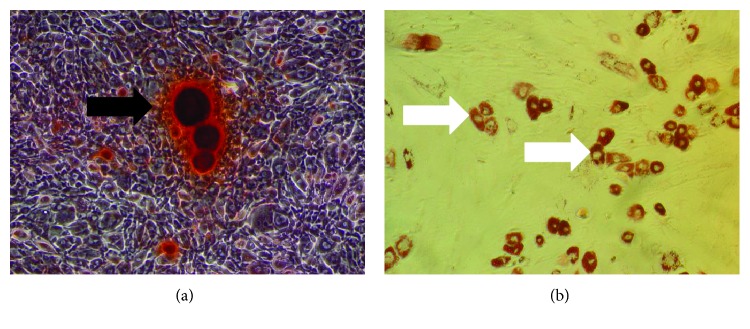
Characterization of rat BMSCs. (a) Alizarin Red S-positive BMSCs. The black arrow indicates the bony nodules. (b) Oil Red O-positive BMSCs. The white arrows indicate lipid droplets.

In order to verify the multiple differentiation potential of the BMSCs, the BMSCs were induced to differentiate into osteoblasts and adipocytes via osteogenic induction and adipocytic induction, respectively. The results of Alizarin Red S and Oil Red O staining are provided in Figure 1. The Alizarin Red S-positive cells are visible in Figure 1(a), where the black arrows indicate the bony nodules. The Oil Red O-positive cells are visible in Figure 1(b), where the white arrows indicate the lipid droplets in the Oil Red O-positive cells.

### 3.2. The Effect of AMI and 2 Gy Radiation on the Proliferation of BMSCs

**Figure 2:**
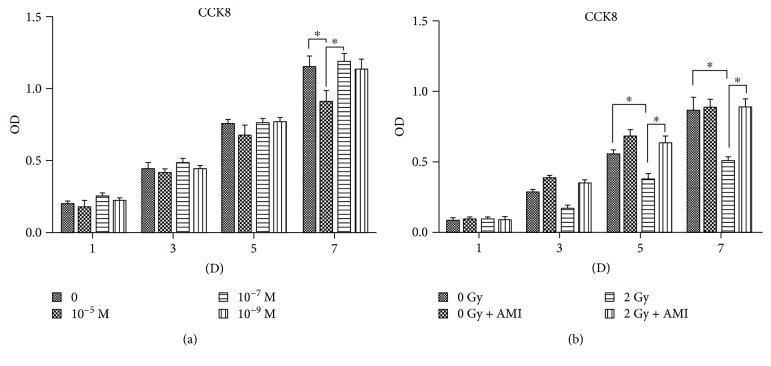
Results of the cell proliferation assay. (a) The effect of different concentrations of AMI on the proliferation of BMSCs. At a concentration of 10^−5^ M, AMI could significantly inhibit the proliferation of BMSCs, in comparison to those after the administration of AMI at concentrations of 0 M and 10^−7^ M, on the seventh day. (b) The effect of AMI and radiation on the proliferation of BMSCs. The proliferation of BMSCs in group C, as measured on the fifth and seventh days, was much lower than that of group A and group D. ^∗^*P* < 0.05. All the values are expressed as the mean ± SD; *N* = 5.

As depicted in Figure 2(a), there was no difference in the proliferation of cells treated with 0 M, 10^−7^ M, and 10^−9^ M AMI. However, the proliferation of cells in the group treated with 10^−5^ M AMI was significantly inhibited on the seventh day of the experiment. Therefore, AMI was administered at a concentration of 10^−7^ M in the subsequent experiments.  Figure 2(b) demonstrates that the proliferation of BMSCs in group C was significantly inhibited in comparison to that of group A (*P* < 0.05) on the fifth and seventh days of the experiment. The proliferation of cells on the fifth and seventh days was much higher in the group that received 2 Gy radiation in conjunction with AMI than the group that received 2 Gy radiation alone (*P* < 0.05).

### 3.3. The Effect of Radiation and AMI on Osteogenesis

**Figure 3:**
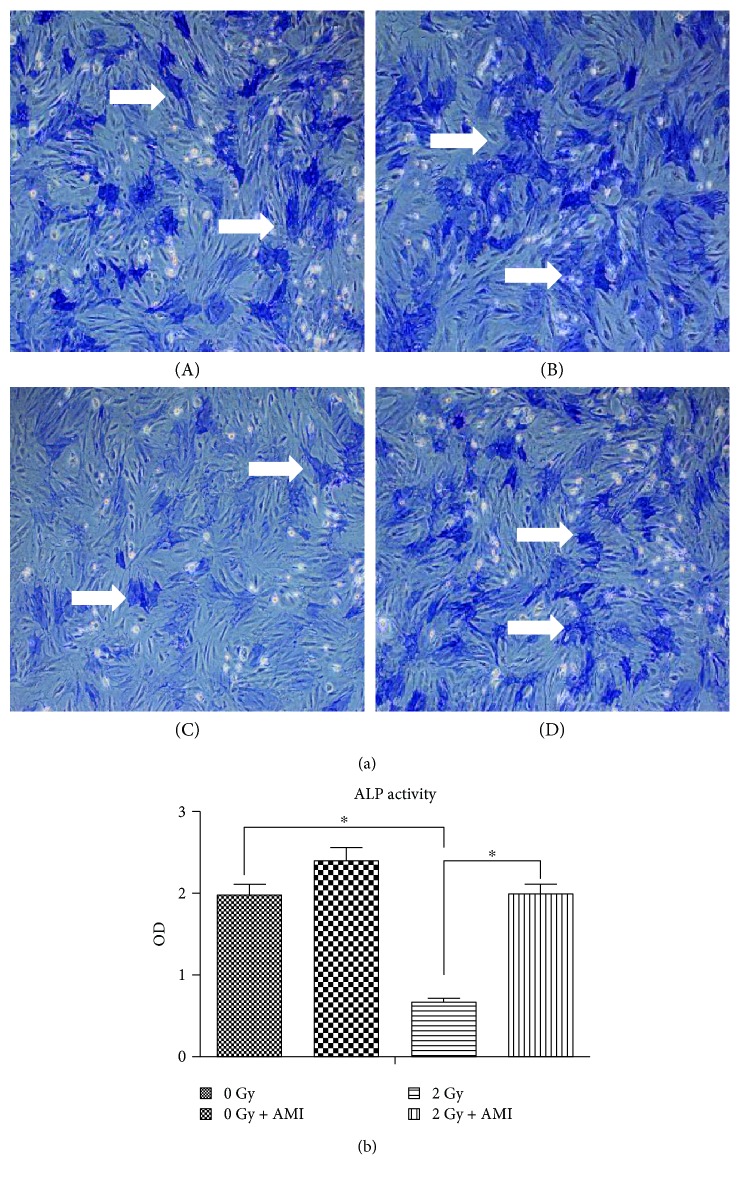
Measurement of ALP Activity. (a) The effect of exposure to 2 Gy radiation and AMI on the activity of ALP in BMSCs. The number of ALP-positive cells, which were of darker color, was higher in group A, group B, and group D than in group C. (b) Statistical data pertaining to ALP activity in the BMSCs. The statistical data indicated that the activity of ALP in group C alone was significantly lower than that in the other three groups. ^∗^*P* < 0.05. All the values are expressed as the mean ± SD; *N* = 5. The white arrows indicate cells positive for ALP activity.

**Figure 4:**
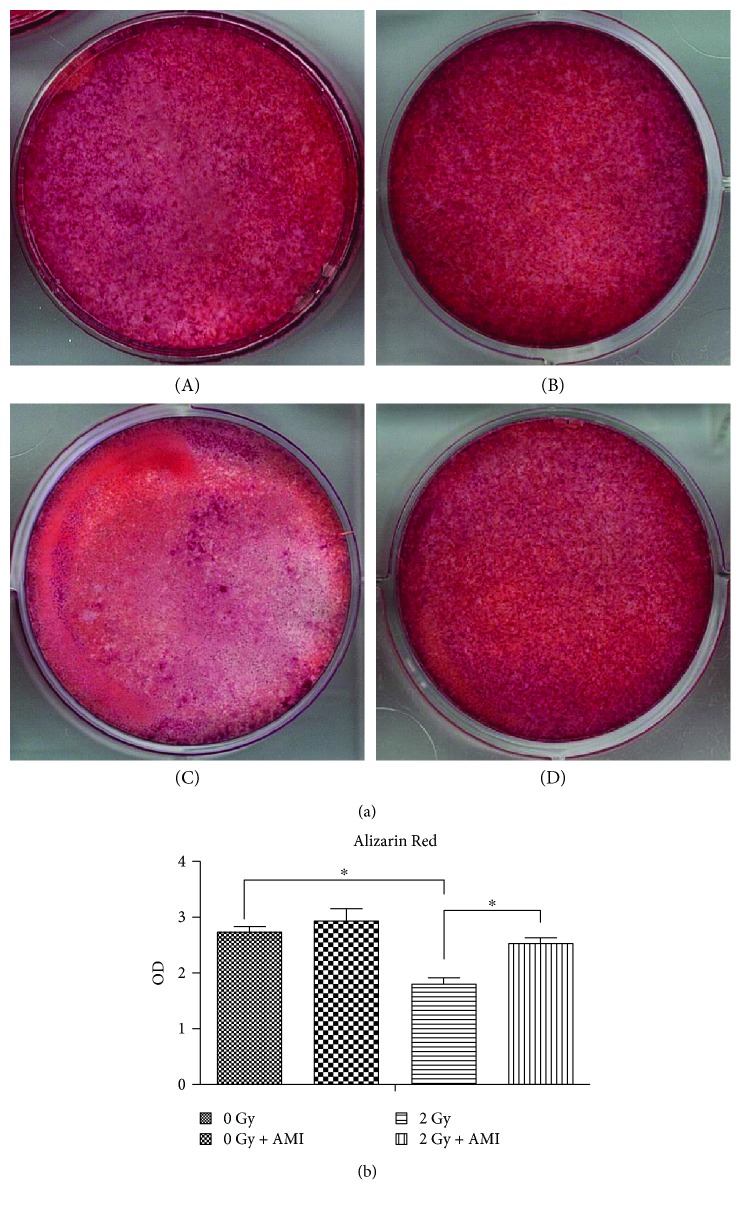
Measurement of calcium deposition. (a) The effect of 2 Gy radiation and AMI on the calcium deposition of BMSCs. The area positive for calcium deposition was much lighter in group C than in the other three groups. (b) Statistical data pertaining to calcium deposition. ^∗^*P* < 0.05. All the values are expressed as the mean ± SD; *N* = 5.

Both ALP activity and calcium deposition were used to assess the effect of AMI and 2 Gy radiation on the osteogenetic differentiation of BMSCs. As depicted in Figure 3(a), the activity of ALP was lower in group C than in group A (*P* < 0.05), with the reduction in the ALP activity being approximately 65% (Figure 3(b)). The activity of ALP was much higher in group D than in group C (*P* < 0.05). However, the difference in ALP activity between group A and group C was not statistically significant. The results of the calcium deposition assay were similar to those of the ALP activity assay (Figures 4(a) and 4(b)).

### 3.4. The Effect of Radiation and AMI on Adipogenesis

**Figure 5:**
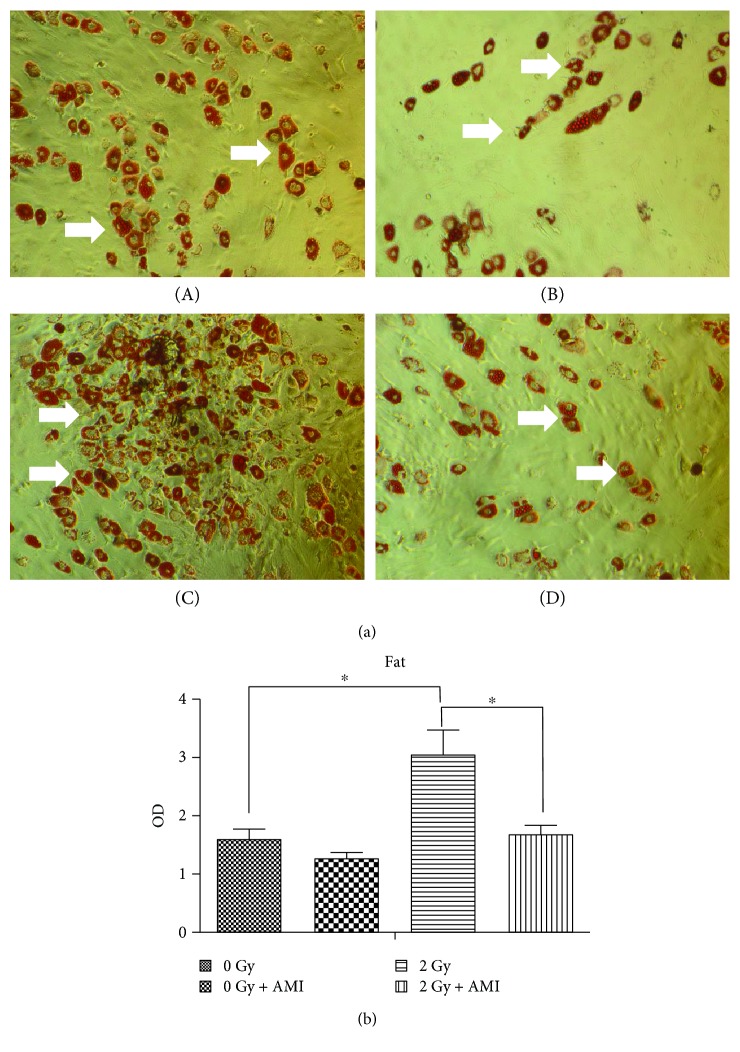
Determination of the adipogenesis of BMSCs. (a) The effect of radiation and AMI on the adipogenesis of BMSCs. The number of cells positive for adipogenesis was higher in group C than in the other three groups. (b) Statistical data pertaining to the adipogenesis of BMSCs. ^∗^*P* < 0.05. All the values are expressed as the mean ± SD; *N* = 5. The white arrows indicate cells positive for adipogenesis.

As depicted in Figure 5(a), a greater number of Oil Red O-positive cells were observed 7 days after radiation in group C than in group A. The quantity of TG in the group subjected to 2 Gy radiation was increased by approximately 110% in comparison to that of the group subjected to 0 Gy radiation (Figure 5(b)). However, the amount of TG in group D decreased by nearly 45% in comparison to that of group C. The amount of TG in group A and group D was almost the same.

### 3.5. The Effect of Radiation and AMI on the Generation of ROS in BMSCs

**Figure 6:**
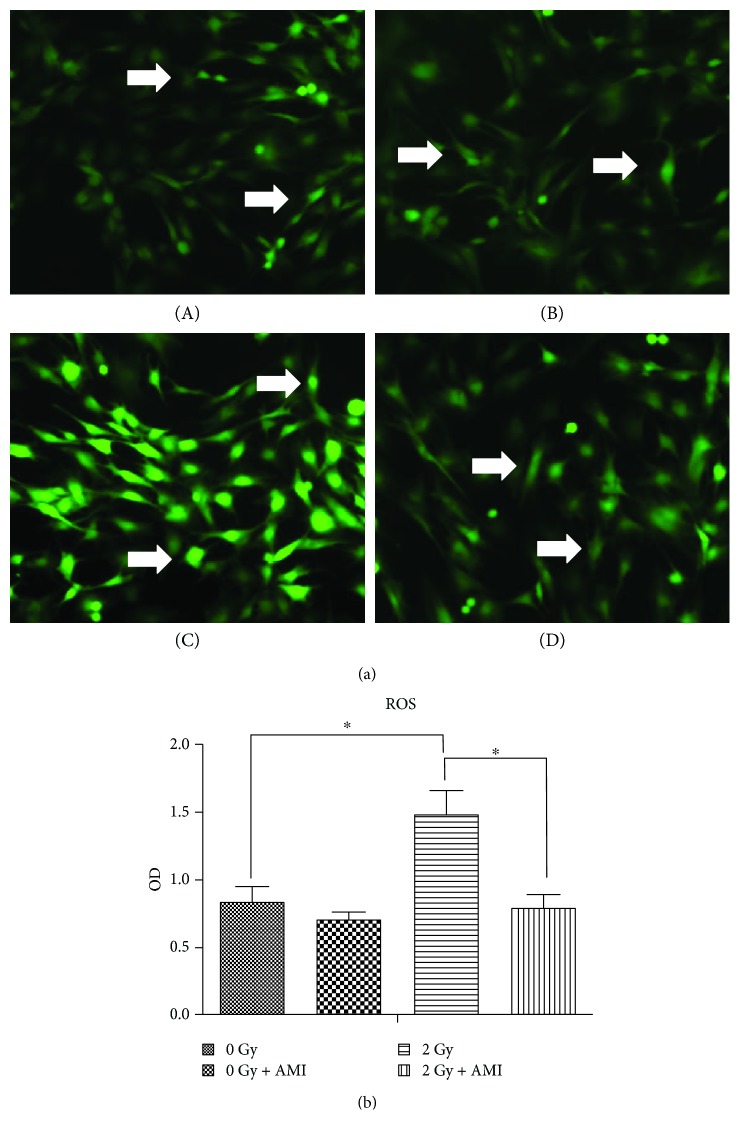
The generation of ROS in the BMSCs. (a) The effect of radiation and AMI on the generation of ROS in the BMSCs. A brighter intensity of green indicated a higher concentration of oxygen free radicals. More ROS positive of higher color intensity were observed in group C, in comparison to those in the other three groups. (b) Statistical data pertaining to the generation of ROS in the BMSCs. The statistical data indicated that the generation of ROS in group C was significantly higher than that in the other three groups. ^∗^*P* < 0.05. All the values are expressed as the mean ± SD; *N* = 5. The white arrows indicate ROS-positive cells.

The levels of ROS in the BMSCs, measured after 2 hours of radiation, increased by approximately 2-fold after exposure to 2 Gy radiation, in comparison to after exposure to 0 Gy radiation (*P* < 0.05). However, the levels of ROS were much lower in group D, in comparison to group C. Additionally, the levels of ROS in group A were similar to those of group D (Figures 6(a) and 6(b)).

### 3.6. The Effect of Radiation and AMI on Apoptosis

**Figure 7:**
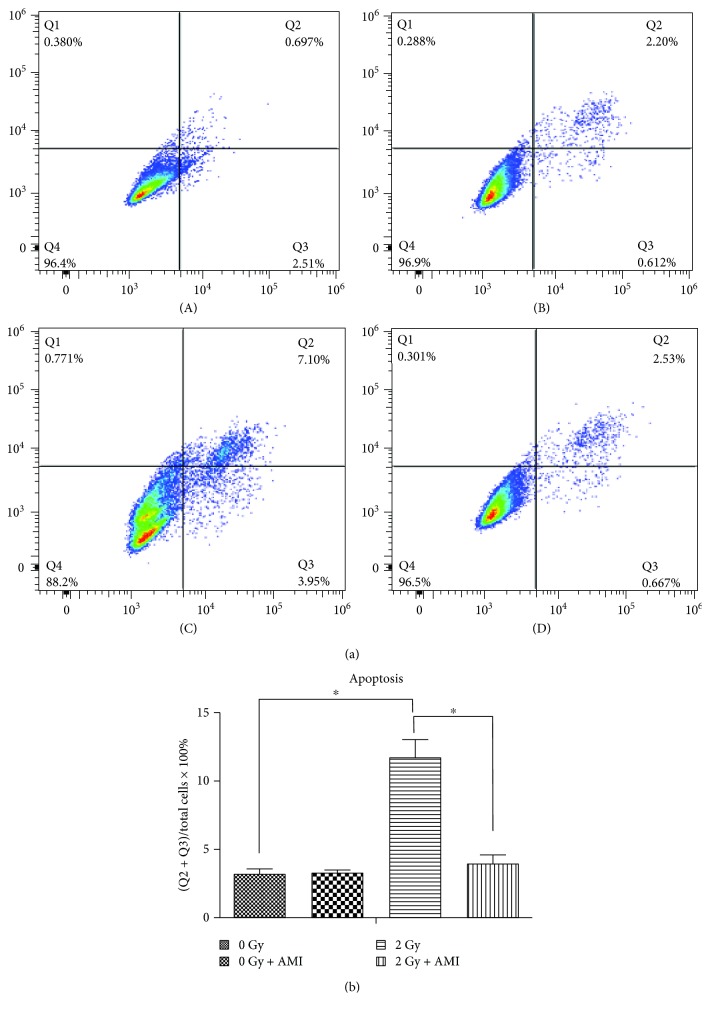
Results of the apoptosis assay. (a) Annexin V-FITC/PI double staining by flow cytometry. (b) Statistical analyses of the data obtained from the apoptosis assay. The number of apoptotic cells was calculated as the sum of Q2 and Q3 (*P* < 0.05; *N* = 4) (Q1, PI-positive and annexin-negative; Q2, both annexin- and PI-positive; Q3, annexin-positive and PI-negative, and Q4, both annexin- and PI-negative).

In order to determine the extent of apoptosis induced by radiation and AMI, an Annexin V/PI staining assay was performed (Figure 7(a)). The percentage of apoptotic cells (Q2 + Q3) in group A, group B, group C, and group D was 3.19%, 3.31%, 11.76%, and 3.96%, respectively (Figure 7(b)), while the percentage of necrotic/dead cells (Q1) was similar among the four groups.

### 3.7. The Effect of Radiation and AMI on the Cell Cycle

**Figure 8:**
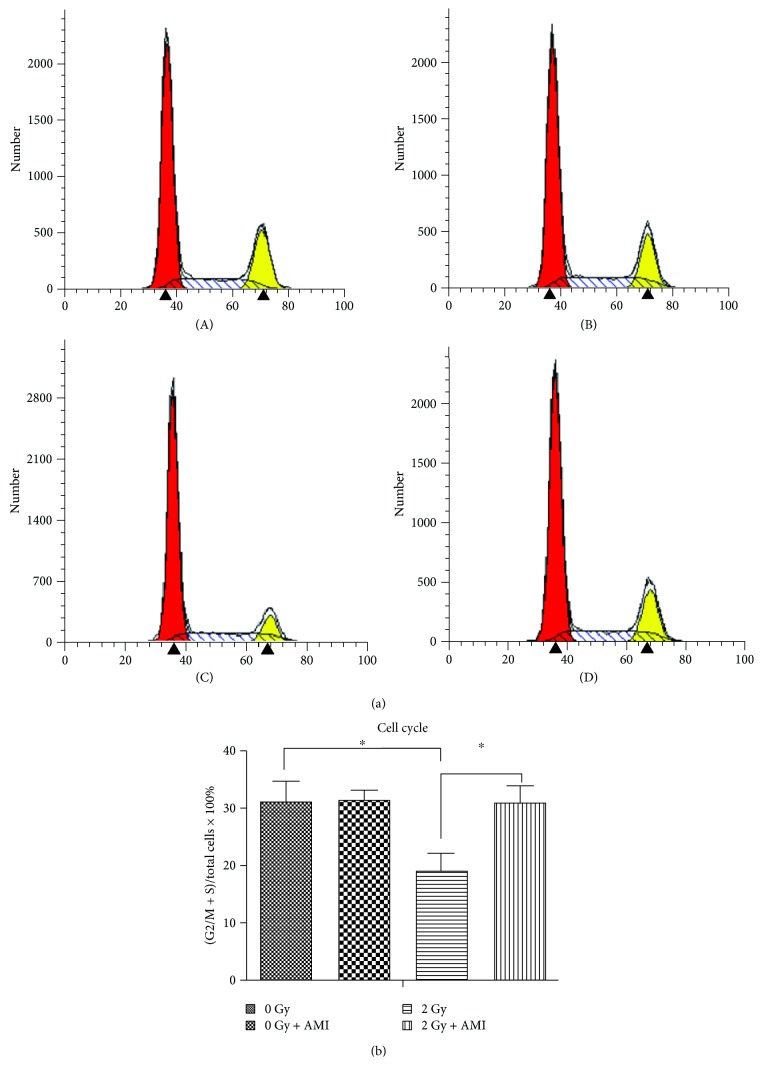
Analysis of the cell cycle of the BMSCs. (a) The effect of radiation and AMI on the cell cycle. (b) Statistical analysis of the cell cycle. ^∗^*P* < 0.05. All the values are expressed as the mean ± SD; *N* = 5.

The results of flow cytometry are depicted in Figure 8(a), and the statistical data are represented in Figure 8(b). The (S + G2) phase accounted for approximately 31.18%, 31.28%, 19.15%, and 30.85% in group A, group B, group C, and group D, respectively. The results demonstrated that cell proliferation was significantly inhibited at a dose of 2 Gy, in comparison to the proliferation of cells subjected to 0 Gy radiation (*P* < 0.05). It was also observed that AMI significantly promoted the proliferation of cells in group D, in comparison to group C (*P* < 0.05).

### 3.8. The Effect of AMI and Radiation on Cell Adhesion

**Figure 9:**
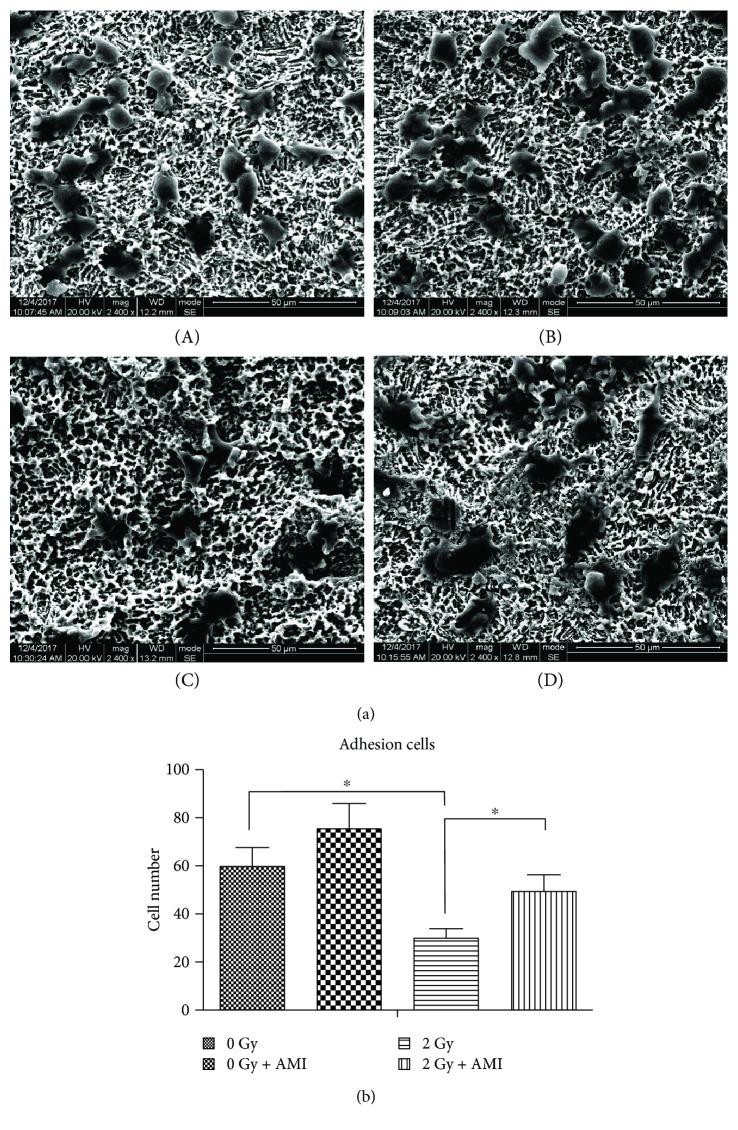
Analyses of cell adhesion. (a) The effect of radiation and AMI on the adhesion of BMSCs. The number of adhesive cells observed in group C was fewer than that in the other three groups. (b) Statistical analysis of the number of adhesive cells. The number of adhesive cells in group C was much lower than that in the other three groups. ^∗^*P* < 0.05. All the values are expressed as the mean ± SD; *N* = 5.

The effect of AMI and radiation on cell adhesion was studied by an SEM (Figure 9(a)). A greater number of cells were observed in group A, group B, and group D, in comparison to group C, with the cells in the three former groups appearing to be more stereoscopic (Figure 9(b)). The number of adhesive cells in group C was strikingly lower than that in group A, group B, and group D (*P* < 0.05).

### 3.9. Western Blot Analysis

**Figure 10:**
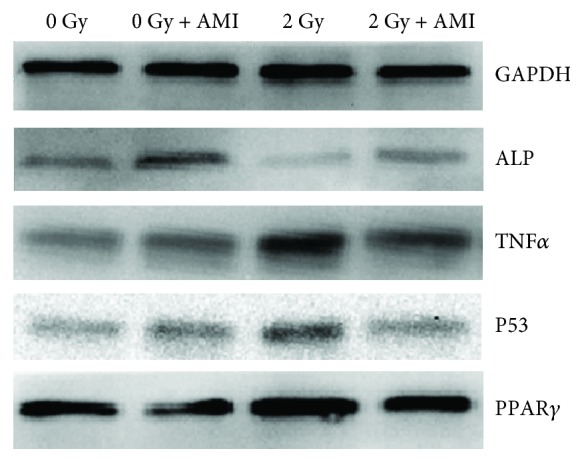
Western blot analysis. The effect of AMI and 2 Gy radiation on the protein expression of ALP, PPAR*γ*, TNF*α*, and P53, as measured 5 days after radiation.

The expression of the proteins related to osteogenesis (ALP), inflammation (TNF*α*), apoptosis (P53), and adipogenesis (PPAR*γ*) was examined 5 days after radiation. As depicted in Figure 10, the expression of ALP was much lower in group C than in group A. However, the expression of TNF*α* and P53 was much higher in group C, in comparison to group A. In both groups, the expression of PPAR*γ* was the reverse of that of the proteins involved in osteogenesis. The expression of ALP was much higher in group D than in group C. However, the expression of TNF*α*, PPAR*γ*, and P53 was much lower in group D than in group C.

## 4. Discussion

Radiotherapy is an effective strategy for the treatment of most solid tumors. Approximately 50% of cancer patients receive radiotherapy at doses of 50–70 Gy [21, 22]. In the clinical scenario, radiation is typically administered in fractions of 2 Gy, and *in vivo* and *in vitro* studies generally use doses of 2 Gy for experimentation [23–25]. Previous studies have demonstrated that when the dose of radiation exceeds 4 Gy, cell proliferation is significantly inhibited by the incident radiation [26, 27]. Therefore, a dose of 2 Gy is typically employed as the study dose, and this dose evidently inhibits the proliferation of cells. However, the study by Nicolay and coworkers revealed that radiation at doses as high as even 9 Gy or 10 Gy does not have any inhibitory effects on the proliferative ability of human primary MSCs [28].

Radiation-induced cell damage arises due to the energy deposited directly onto DNA, leading to DNA double-strand breaks (DSBs), which is one of the most toxic lesions in the genome. It has been reported that 5% of DSBs are irreparable. Unrepaired DSBs can lead to cell death via multiple pathways, including apoptosis, senescence, mutation, or genomic instability [29–32]. Radiation-induced DNA damage can induce the ROS-mediated activation of p38 mitogen-activated protein kinase (MAPK). ROS, which is produced by the radiolysis of water, activates the transcription factor nuclear factor kappa-B, which in turn enhances the expression of p16INK4A. The p16INK4A protein expresses and activates the pRb tumor suppressor protein, which suppresses the expression of certain genes involved in cell proliferation, ultimately leading to durable cell cycle arrest [34, 35].

Apart from activating p16/Rb, DNA DSBs also activate the P53 tumor suppressor protein, which in turn induces the transcription of the p21 WAF1 gene and ultimately causes senescence and permanent growth arrest. Both the p53/p21 and p16INK4a/pRb pathways are crucial for senescence and cell cycle. In order to ensure that damaged DNA is repaired prior to division, the DNA damage checkpoints in the cell cycle are located late in the first gap (G1) phase, which prevents entry to the division (S + G2/M) phase [32, 33, 35–37]. The results of this study demonstrated that radiation administered at a dose of 2 Gy significantly increased the intracellular levels of ROS (Figure 6), while significantly decreasing the number of cells in the division (S + G2/M) phase and significantly increasing the population of cells in the first gap (G1) phase (Figure 8). Our study also demonstrated that the number of apoptotic cells in the group that was exposed to 2 Gy radiation was significantly higher than that in the group that was not exposed to radiation (Figure 7).

However, the administration of AMI to the group that was exposed to 2 Gy radiation significantly increased the number of cells in the division (S + G2/M) phase and significantly decreased the number of cells in the first gap (G1) phase, in comparison to that of the group that was exposed to 2 Gy radiation alone. The number of apoptotic cells in the group that received 2 Gy radiation and AMI was evidently lower than that in the group exposed to 2 Gy radiation only. This indicated that AMI reduced the effect of radiation on the cell cycle and apoptosis. AMI is rapidly dephosphorylated by ALP on the surface of normal cells to its clinically active metabolite, WR-1065 [16, 17]. When activated, AMI protects normal cells from radiation-induced DNA damages by eliminating the oxygen free radicals produced by radiation, which prevents the DNA damage that is induced by oxygen free radicals, and also by donating hydrogens that repair the existing damaged DNA [14–17]. Recent studies have demonstrated that WR-1065 is rapidly oxidized to a polyamine-like disulfide metabolite, WR-33278. During this process, the oxygen in the culture medium is rapidly depleted. The metabolite, WR-33278, upregulates the expression of proteins that are involved in DNA repair and cell cycle regulation, as well as proteins related to apoptosis under hypoxic conditions, such as the hypoxia-inducible factor-1*α*, P53, and Bcl-2, which can initiate or inhibit apoptosis according to the cell type-specific or cell status-specific interactions [17, 38, 39]. Previous studies have demonstrated that AMI upregulates cell proliferation in the early phases of injury following radiation therapy and also enhances the rate of cell survival [23, 40].

Previous studies have reported that BMSCs are sensitive to radiation in both *in vivo* and *in vitro* conditions and that the osteogenic differentiation and adipogenic differentiation of BMSCs are reciprocal to one another [41]. Our study demonstrated that while radiation inhibited the osteogenic differentiation of BMSCs, it promoted their adipogenic differentiation, which was similar to the results of the previous study [42]. The critical nuclear receptor protein, PPAR*γ*, which is related to lipogenesis and is essential for the differentiation of adipocytes, was measured on the 5th day after radiation. PPAR*γ* regulates the balance between the osteogenic differentiation and adipogenic differentiation of BMSCs. The results of this study suggested that in comparison to that of normal cells, the expression of PPAR*γ* increased in the group that was exposed to 2 Gy radiation, but the levels of PPAR*γ* decreased to normal when AMI was added [43]. Furthermore, it has been previously demonstrated in an *in vivo* study that exposure to radiation enhances the formation of adipose tissue in the bone marrows, with the bone marrows being partially replaced by adipose tissues in irradiated bones [44]. However, both ALP activity and calcium deposition were much higher in the group that received 2 Gy radiation and AMI, in comparison to the group that received 2 Gy radiation alone. A study by Wong and coworkers reported that AMI could prevent the radiation-induced suppression of normal osteoblastic differentiation and pretreatment with AMI results in a statistically significant increase in ALP activity, compared to irradiated cells [40]. Some other studies have reported that AMI, in combination with radiation therapy, can enhance the activity and expression of ALP as well as the expression of the genes related to osteogenesis. No significant differences was reported in the activity and expression of ALP or the expression of the osteocalcin gene, between the group that was exposed to radiation and the group that received the radioprotector in combination with radiation [23, 40, 45]. The microenvironment of tumor tissues and tumor cells is weakly acidic. The activity of ALP in this environment is low, and the activity of acid phosphatase is high, in comparison to normal cells. Although AMI can be activated by ALP, it is not activated by acid phosphatase. Therefore, the activation of AMI in tumor tissues or tumor cells is difficult. On the other hand, the microenvironment of normal tissues is weakly alkaline. The activity of ALP in normal cells is high while the activity of acid phosphatase is low in comparison to tumor cells, which makes the activation of AMI in normal tissues much easier than in tumor cells [12–15].

Furthermore, several *in vivo* studies have demonstrated that AMI plays a crucial role in protecting bones from the scourge of radiation-induced complications. This study demonstrated that the depolarization ratios of mineral to collagen were significantly lower in the group that received radiotherapy than in the group that received radiotherapy in conjunction with AMI. Studies using Raman spectroscopy have reported that radiation induces damages to the chemical composition and ultrastructure of bone and prophylaxis with AMI causes a recovery towards the normal, native composition and orientation of the minerals and collagen in bones [46, 47]. In a rat model of radiotherapy with mandibular distraction osteogenesis, the mean bone volume fraction significantly decreased in the group that received radiotherapy alone in comparison to the group that received radiotherapy in conjunction with AMI (0.35 vs. 0.76). The study further reported that the mean bone mineral density (mg/cc) was also significantly reduced by 51.6% in the fracture group that received radiotherapy alone, in comparison to the group that received a combination of radiotherapy and AMI [12, 47]. Another study reported that the region of union in the group that received radiotherapy in conjunction with AMI was three times as large as that of the group that received radiotherapy alone and that the mean bone break loading for the radiated and distracted groups was significantly lower (34.77 ± 30.78 N) than that of the group pretreated with AMI (61.74 ± 48.49 N) [48]. Besides, when administered prior to radiotherapy, AMI can protect the vascular network by maintaining the caliber of the blood vessels, such that they are comparable to those of normal, nonirradiated bones. AMI can significantly increase the thickness and vascular volume fraction of blood vessels in irradiated bones [12, 49].

## 5. Conclusion

This study is a first of its kind to investigate the effect of AMI on BMSCs in a rat model of radiation therapy. This study provides quantitative evidence regarding the cellular injury caused by irradiation and demonstrates its adverse effects, while highlighting the beneficial effects of AMI on BMSCs. It was observed that AMI could promote the osteogenic differentiation potential of BMSCs that were exposed to 2 Gy radiation, by eliminating the oxygen free radicals produced by the incident radiation and by promoting the proliferation of BMSCs while suppressing their adipogenic differentiation potential. At present, AMI is the only radioprotective drug that has approval for clinical use and our work provides a basis for the clinical evaluation of AMI in alleviating radiation-induced injury.

## Data Availability

The data underlying the findings of this study are available at https://figshare.com/articles/Amifostine_reduces_the_effect_of_radiation_on_BMSCs_by_promoting_cell_proliferation_and_reducing_ROS/6870725.
